# The Transport of Territorial Field Medical Units

**Published:** 1909-12-18

**Authors:** 


### The Hospital

A Journal of

The Medical Sciences and Hospital Administration.

New Series. No. 147, Vol. VI. [No. 1219, Vol. XLVII.] SATURDAY, DECEMBER 18, 1909.

### The Transport of Territorial Field Medical Units.

On a subsequent page of this issue will be found a communication on the Transport of Territorial Field Ambulances, to which the particular attention of the profession may be most seriously invited. The authorship of this article we are not at liberty to disclose, but the authority and sincerity which stamp every word of it would be, if possible, enhanced were we to do so. The case against the War Office is there stated with such moderation that the impression may gain ground that on the whole things are well with the Territorial Army, or at least with its medical branches. That impression, in our deliberate judgment, is very far indeed from representing the facts: and it may be useful, in the interregnum before a General Election, to indicate some of the matters which demand the immediate consideration of the next Secretary of State for War.

On the question of the transport of Field Ambulances our correspondent's facts are incontrovertible, and they reveal a very serious state indeed. At the last meeting of the British Medical Association Colonel Giles, the A.M.O. of the First London Division (T.F.), read a paper on this very subject, which obtained the support of all territorial medical officers (*British Medical Journal*, August 4, 1909, p. 385). His very sensible suggestions have been ignored; most of his cardinal principles have been violated. When, two years and more ago, Sir A. Keogh stumped the country, appealing to the profession to join the R.A.M.C. (T.) and to make the new organisation a success, it was felt that he had abandoned the old traditions of Whitehall, and that at last and at least one branch of the auxiliary forces was to be fairly treated. The original regulations were drawn up on a scale which seemed to warrant this assumption, and the profession responded to the appeal by filling up the (commissioned) ranks of the Field Ambulances. Of the good points of the scheme the best of all—an advance, by the way, on the conditions in the Regular Army—was that by which medical units were to enlist and train their own transport, and the transport equipment was provided on an adequate scale. Now, this scale is cut down: such few waggons as are to be issued will have no harness, and civilian waggons and harness are to be hired for annual training. Lucky units to which the full equipment had been issued, or which possessed it as a legacy from Volunteer predecessors, have had to hand it back. The original scheme may be supposed to have been advised by responsible officers, of whom the Director-General must have been one. If that scale represented the minimum compatible with real war efficiency (and no one would dream of regarding it as anything more than the minimum), then the new one permits only the most pitiable inefficiency, and those who have consented to it, the Director-General included, have failed most signally in their duty. The Field Ambulances and Mounted Brigade Ambulances *on paper* are complete units ready for war: in actual fact they have hardly any waggons (some of them have none at all so far), they have no harness, no horses (and an insufficient allowance even of those hired for camp purposes), and inadequate stores. Many of the waggons they hire for training purposes are quite useless for the purposes to which they are put, and some of them are actually dangerous, as was seen on Salisbury Plain this year.

But there are many other grievances. The men of the transport sections are paid a penny a day less than their comrades of the bearer and tent division: yet they have to learn their transport duties in addition to ambulance work, at which latter they must attend forty-two drills. No transport officer is allowed by the regulations, but one of the (medical) officers is to undertake the duties and to have his pay cut down three or four shillings a day in consequence, if a somewhat obscure passage in the regulations be interpreted in the usual Treasury spirit. No quartermaster and no adjutant are provided. It is the duty of County Associations to provide proper headquarters for these units, without which, as any Volunteer officer knows, it is impossible to maintain efficiency, numbers, discipline, or anything else that spells success. Yet in London, which ought surely to set an example to the rest of the country, some units are still without headquarters, and others are so housed that a motion of indignation and protest on the matter was put down for the last meeting of the County Association. The latest news is that, after the refusal of more than one excellent proposal by the officials at Whitehall, the Association is practically compelled to plant several of these units in the middle of a slum which is neither central nor easily reached, and is a hotbed of crime and vice.

The messing allowance for camp is not generous enough; those young men who give up part of their scanty leisure to attend camp deserve rather more of their country than semi-starvation. An allowance per head which will, with the best management, just suffice for a battalion of 800 or 1,000 men will not do the same for a Mounted Brigade Ambulance of 102: this fact has only to be stated to be obvious. Again, the Mounted Brigade Ambulances, even on their original basis, were not nearly mobile enough; it is quite impossible, therefore, that on their present reduced establishment they can ever keep up with mounted troops. There is but one spare draught horse for a whole ambulance, and only three of the non-commissioned ranks are allowed riding horses. The Field Ambulances have no spare draught horse at all. Lastly, there is a grievance which affects all territorial officers equally. It is the deduction of income-tax from their pay whilst in camp. Unless, indeed, they send in a full return of their income from all sources, the Army Pay Department assumes that they have £2,000 a year, and deducts the maximum tax accordingly. But, apart from that, the expenses of attending training in camp amount to much more than the pay allowed by regulation for it; and to reduce this scanty sum by deducting income-tax from it is to treat those who are devoting their time to their country's service in that same spirit of contemptible meanness which distinguished the old Volunteer days, and was supposed to be abandoned at the advent of the new era.

